# Applications of Quantitative Systems Pharmacology (QSP) in Drug Development for NAFLD and NASH and Its Regulatory Application

**DOI:** 10.1007/s11095-022-03295-x

**Published:** 2022-05-24

**Authors:** Scott Q. Siler

**Affiliations:** DILIsym Services, a Division of Simulations Plus, 510-862-6027, 6 Davis Drive, PO Box 12317, Research Triangle Park, North Carolina 27709 USA

**Keywords:** fibrosis, modeling, NASH, QSP

## Abstract

Nonalcoholic steatohepatitis (NASH) is a widely prevalent disease, but approved pharmaceutical treatments are not available. As such, there is great activity within the pharmaceutical industry to accelerate drug development in this area and improve the quality of life and reduce mortality for NASH patients. The use of quantitative systems pharmacology (QSP) can help make this overall process more efficient. This mechanism-based mathematical modeling approach describes both the pathophysiology of a disease and how pharmacological interventions can modify pathophysiologic mechanisms. Multiple capabilities are provided by QSP modeling, including the use of model predictions to optimize clinical studies. The use of this approach has grown over the last 20 years, motivating discussions between modelers and regulators to agree upon methodologic standards. These include model transparency, documentation, and inclusion of clinical pharmacodynamic biomarkers. Several QSP models have been developed that describe NASH pathophysiology to varying extents. One specific application of NAFLDsym, a QSP model of NASH, is described in this manuscript. Simulations were performed to help understand if patient behaviors could help explain the relatively high rate of fibrosis stage reductions in placebo cohorts. Simulated food intake and body weight fluctuated periodically over time. The relatively slow turnover of liver collagen allowed persistent reductions in predicted fibrosis stage despite return to baseline for liver fat, plasma ALT, and the NAFLD activity score. Mechanistic insights such as this that have been derived from QSP models can help expedite the development of safe and effective treatments for NASH patients.

## Manuscript

Non-alcoholic fatty liver disease (NAFLD) and non-alcoholic steatohepatitis (NASH) are diseases of the liver that are largely the result of excessive lipid accumulation and partitioning (1, 2). The incidence of NAFLD and NASH have grown substantially over the last 20 years, with estimates of 20–40% of populations in various locations throughout the world having NAFLD or NASH (3). As such, the interest in understanding and treating this disease has increased substantially. NASH is reasonably well-understood, with the overall understanding growing every year. The pathophysiology includes contributions from alterations in lipid partitioning, lipotoxicity, inflammation, and fibrosis (1, 2). Data from clinical studies with the numerous potential treatments have helped increase the overall understanding of the disease in addition to focused clinical studies.

NASH drug development efforts have increased substantially in the last 10 years. A considerable number of potential treatments are being developed across the pharmaceutical industry (4, 5). In addition to monotherapies, several compounds are being developed as combination treatments; complimentary mechanisms of action may provide additional clinical benefit. Unfortunately, the development of several compounds has been terminated (6). This has primarily been due to an insufficient ability to demonstrate favorable responses in treatment cohorts as compared with placebo cohorts.

Regulators want to ensure that NASH drugs being developed are both safe and effective. The United States’ Food and Drug Administration has provided definitions of endpoints that have the greatest chance of providing health benefits of patients (7). Data are not yet available identifying ideal surrogate endpoints for preventing adverse clinical outcomes. Measures derived from liver biopsies are currently employed to characterize resolution of NASH (based on specific histologic endpoints related to pathophysiologic components) as well as improvements in hepatic fibrosis (8, 9).

Efficient drug development will benefit NASH patients by minimizing the period of time for them to gain access to safe and effective medicines. Application of mathematical modeling practices such as quantitative systems pharmacology (QSP) can expedite clinical drug development (10). QSP modeling is a mechanism-based mathematical modeling approach that describes not only the pathophysiology of a disease, but also how pharmacological interventions can modify the mechanisms of pathophysiology (11–15). Simulated populations (aka virtual populations) provide a useful approach for capturing the pathophysiology of the disease inasmuch as they provide the ability to make predictions that account for inter-patient variability in both disease pathophysiology and clinical status (13, 14, 16). Predicted pharmacologic effects on simulated patients result from predicting compound exposure and pharmacodynamics (PD). Compound exposure at the site of the target can be predicted by the use of physiologically-based, pharmacokinetic (PBPK) modeling; this is particularly valuable when simulating pharmacologic intervention of intracellular targets. The PD and/or mechanism of action (MoA) of compounds can be simulated by translating laboratory and/or clinical data into equations that describe how existing pathophysiologic processes are altered by the actions of the compound.

QSP is a relatively new approach that has historical origins (17). Technological advances in computer chip design provided the ability for simulations results to be generated in much shorter periods of time, enabling this modeling approach to be applied to pharmaceutical drug development. The use of QSP modeling continues to grow (18, 19). Capabilities provided by QSP modeling include predicting efficacy, identifying responsive patient types, determining appropriate trial duration and measurement timing, delineating placebo response from pharmacologic response, understanding the link between mechanisms and biomarker responses, and predicting the efficacy potential for combination treatments (12, 20, 21). Simulation results of these sorts can inform clinical trial protocol design.

NAFLD and NASH provide some unique considerations with respect to QSP modeling. The pathophysiology includes several discrete, yet interactive areas: steatosis, lipotoxicity, inflammation, and fibrosis. QSP models of NAFLD and NASH should include representations of each of these areas, as the response to treatments usually invoke responses from each. Within the steatosis area, the uptake and/or de novo synthesis of fatty acids as well as esterification into triacylglycerol (TG) are primary components. Lipotoxicity is characterized by an excess of lipids that ultimately leads to hepatocellular apoptosis. Inflammation in NASH includes the recruitment of additional immune cells to the liver as well as increased production of key immune mediators. Fibrosis is characterized both by the presence of excessive amounts of collagen as well as the activated hepatic stellate cells that produce the extracellular matrix components. QSP models of NASH should include equations that describe these pathophysiologic mechanisms in addition to the primary biomarkers (e.g., plasma ALT) and histologic outputs that are used to determine efficacy.

QSP models of NASH also need to represent the various patient types that are recruited into NASH clinical studies. This includes overweight or obese individuals that have not had a diagnosis of NASH for Phase I studies; these patients may or may not have steatosis, and are unlikely to have much lipotoxicity, inflammation, and/or fibrosis. Phase IIa studies typically include patients with NASH and varying degrees of fibrosis. Phase IIb and Phase III studies typically recruit patients with stage 3 or 4 fibrosis in addition to NASH, as documented by liver histology. Simulated cohorts with each of these patient types will enable a NASH QSP model to support development throughout the lifecycle of the compound.

The awareness of QSP modeling within regulatory agencies such as the FDA has grown as the modeling approach has been increasingly utilized. Currently, the FDA has not issued any direct guidance about NASH QSP modeling, but the agency has indicated that there are certain recommendations that should be applied to all QSP modeling (18, 22). Model transparency enables reviewers to have access to all equations and parameters used to generate simulation results. Not only will it enable reviewers to recapitulate simulation results, model transparency will also allow reviewers to interpret simulation results. Similarly, documentation of the modeling rationale facilitates this process. Another recommendation from FDA about QSP modeling is for model developers to submit a minimum model. Reducing the complexity of the model further enables reviewers to better interpret simulation results. Similarly, prespecified quantitative or statistical criteria helps reviewers determine the validity of the predicted results. Finally, the FDA has recommended that there be maximal inclusion of clinical PD markers. Applying this recommendation to NASH indicates that the ability for the QSP model to predict changes to the histological measurements, NAFLD Activity Score (NAS) and fibrosis stage, is crucial; these are the currently acceptable endpoints for efficacy for NASH treatments (7). Additional biomarkers such as liver fat, plasma ALT, and serum Pro-C3 biomarkers have been routinely employed in NASH clinical studies; their inclusion in a NASH QSP model further enhances its utility.

An additional utility of a NASH QSP model would be the ability to reproduce the key results of clinical studies. This includes not only the response to the investigational treatment but also the response of the placebo group. The lack of separation between the treatment and placebo cohorts has been a significant reason for the termination of a number of promising potential treatments for NASH (6). In particular the fraction of patients within the placebo cohorts that show improvement in fibrosis stage over 26, 48, or 72 week studies is surprisingly high (20–30%, (23)). There is some indication that operator variability in the reading of the histological liver biopsy samples may play a significant role (24), and there may be contributions from other factors as well.

Several useful QSP models of hepatic steatosis, hepatic fibrosis, and NAFLD/NASH have been reported. Ashworth *et al*. developed a QSP model of lipid partitioning and steatosis, including zonal differences across the hepatic acinus (25). Liao *et al*. extended this model to include the effects of fructose on lipid partitioning and hepatic steatosis (26). Neither of these QSP models included representations of lipotoxicity, inflammation, fibrosis, or NASH biomarkers or the primary histologic endpoints of NAS and stage of fibrosis. Holzhutter and Berndt also developed a QSP model of zonal influences on hepatic steatosis (27); this model also includes hepatocellular death as a consequence of lipid accumulation. It does not, however, include representations of inflammation, fibrosis, or NASH biomarkers. Dutta-Moscato *et al*. developed an agent-based QSP model of hepatic fibrosis, but it is not specific to NASH (28).

A more comprehensive QSP model of NAFLD and NASH is NAFLDsym (29–33). This QSP model includes interactive sub-models of steatosis, lipotoxicity, inflammation, and fibrosis. Several important sub-models, such a hepatocyte death and proliferation, meal administration and post-prandial metabolites, mechanistic representations of alanine aminotransferase (ALT) and aspartate aminotransferase (AST) amongst others, were replicated from the quantitative systems toxicology (QST) model, DILIsym (34, 35). Also replicated from DILIsym are discrete representations of hepatocytes within the periportal, midlobular, and centrilobular sections of the hepatic acinus. NAFLDsym also includes representations of a variety of useful, NASH-specific biomarkers, including histologic NAS and fibrosis stage outputs as well as serum pro-C3. Moreover, there are more than 1700 simulated patients, enabling focused exploration of different subsets of NAFLD and NASH patients (e.g., fibrosis stage 3). These simulated patients have been validated by simultaneous comparison to many clinical data measurements that focus on specific aspects of the pathophysiology. A variety of treatments in clinical development have been simulated with NAFLDsym, including cenicriviroc and anti-FGFR1/KLB bispecific antibody (36, 37). Predictions of improvement with weight loss as well as disease progression with weight gain have also been reported with NAFLDsym (30), providing further validation of the model.

**Fig. 1:**
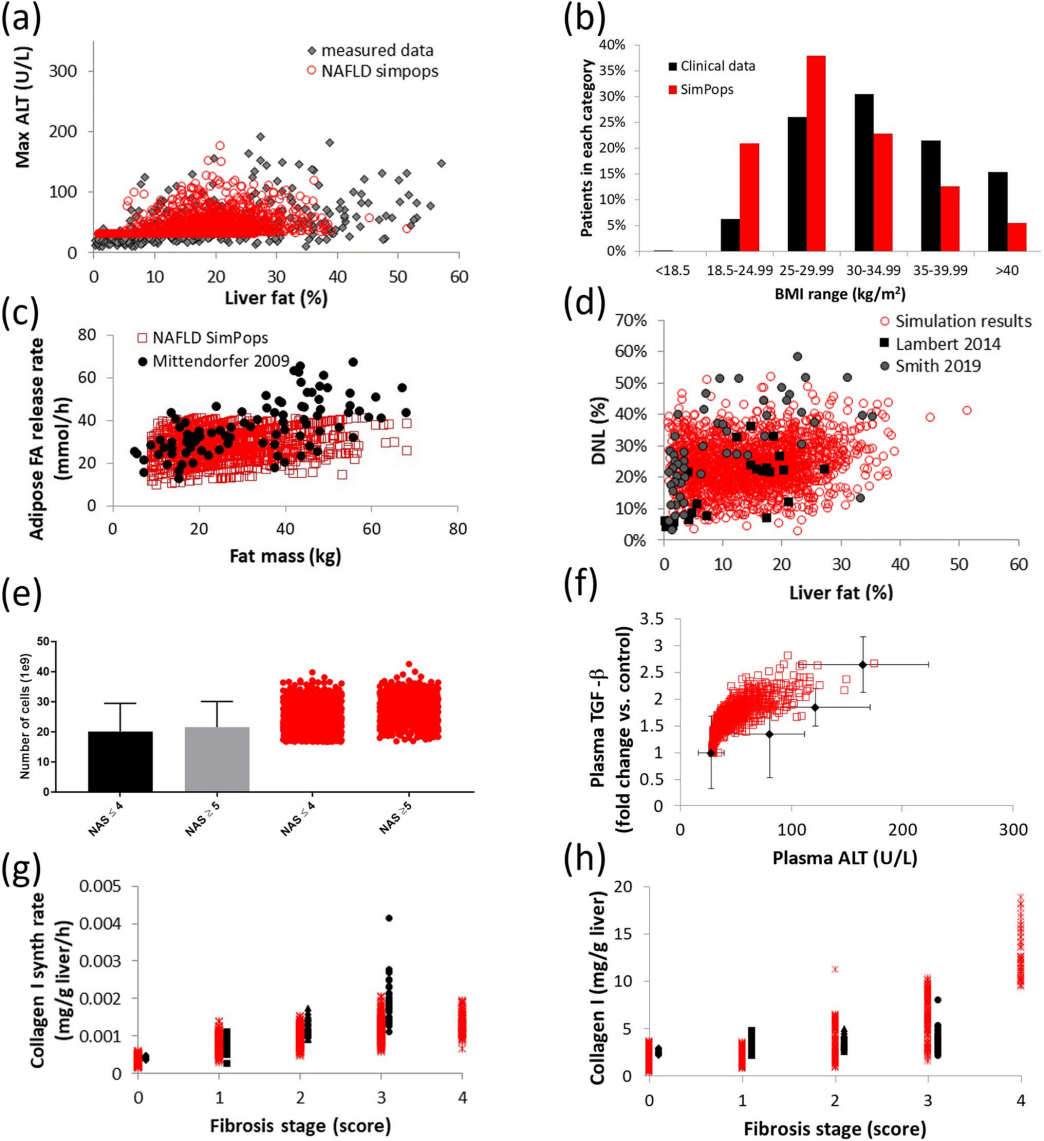
Simulation results and clinical data enabling characterization of sub-model behavior and several important aspects of NASH pathophysiology as simulated by NAFLDsym. The relationship between liver fat and plasma ALT in the simulated patients is quite similar to the measured data from Maximos *et al*. (**a**); the simulated population retains a distribution of BMI that is comparable to the clinical data reported by Dudekala *et al*. (**b**); the relationship between fat mass and adipose fatty acid (FA) release rates is comparable between the simulated patients and the clinical data reported by Mittendorfer *et al*. Note that there are few simulated patients with adipose FA release rates in excess of 50 mmol/h (**c**); the ranges of de novo lipogenesis (DNL) and liver fat are comparable between the simulated patients and the clinical data reported by Lambert *et al*. and Smith *et al*. (**d**); the number of lobular macrophages in NAFLD and NASH patients is consistent with the clinical data reported by Tajiri *et al*. Note that there are minimal differences between patients below or above NAS = 4. (**e**); the range of TGF-beta levels in simulated patients and clinical cohorts with varying plasma ALT levels, as reported by Dal *et al*. (**f**); synthesis rates and quantities of hepatic collagen type I in clinical and simulated patients across a range of fibrosis scores. Clinical data were reported by Decaris *et al*. and Masugi *et al*. Clinical patients with fibrosis stage = 4 were excluded from figure, as data for only two patients were reported. (**g**,**h**). In each figure, black or grey symbols represent clinical data sets while red symbols represent simulated patients. In figures a, c-h, individual simulated patients are displayed.

There are four sub-models within NAFLDsym that particularly address the major elements of NASH pathophysiology: steatosis, lipotoxicity, inflammation, and fibrosis. Concise descriptions of each submodel are given below. Figure 1 also displays several simulation results that characterize some of the key outputs of these sub-models.

The steatosis sub-model within NAFLDsym includes multiple processes essential for the trafficking of fatty acids, diacylglycerols, and TG within hepatocytes. One of these key processes included in NAFLDsym is the uptake of circulating fatty acids from the circulation in a concentration-dependent manner following the release of fatty acids into the circulation by adipocytes (38, 39). Hepatic de novo lipogenesis of fatty acids is also represented within NAFLDsym, including contributions in the overnight-fasted as well as the post-prandial states (40–42); hepatocytes in NAFLDsym take up glucose from the circulation in the post-prandial period, when plasma glucose levels are elevated (43, 44). In NAFLDsym, hepatocytes can also use either intracellular fatty acids or pyruvate to as fuels support the production of ATP to meet bioenergetic demands. The representation of ATP production is quite similar to what has previously been modeled in DILIsym (34, 35). Fatty acid oxidation is regulated by both ATP levels as well as the relative availability of both fatty acids and pyruvate (45). Fatty acids can be esterifed to TG in NAFLDsym; lipolysis of hepatocellular TG is also represented (46). Finally, the export of intracellular TG as part of very-low-density lipoprotein (VLDL-TG) is also represented (47, 48).

Lipotoxicity in NAFLDsym is primarily represented based on the mechanistic interactions between saturated fatty acids (SFA), oxidative stress, and hepatocellular apoptosis (49–51). A variety of clinical data were used to guide the optimization of parameter values within these processes (52–55). Both liver SFA and unsaturated fatty acids (UFA) are separately tracked within NAFLDsym. The accumulation of hepatocellular SFA elicits an increase in reactive oxygen species (49–51). The resultant cellular oxidative stress, in sufficient quantities, yields hepatocellular apoptosis; necrosis can occur at very high levels of oxidative stress. However, circulating biomarkers such as cleaved cytokeratin 19 (cK18) and histological ballooning indicate that apoptosis predominates (55, 56). The magnitude of hepatocyte loss due to lipotoxicity can be estimated by computational methods (57), providing validation of the steady state numbers of viable hepatocytes in the simulated NASH patients. Apoptotic hepatocytes release vesicles that contain cellular fragments. These vesicles and the fragments within them can interact with Kupffer cells, macrophages, neutrophils, and other immune cells to provide a degree of activation of the immune system (58–60).

The inflammation sub-model in NAFLDsym includes resident Kupffer cells as well as recruited macrophages and neutrophils. The numbers of each cell type in the simulated patients have been calibrated to be consistent with clinical data (61, 62). Interestingly, the numbers of macrophages and neutrophils do not vary much between NASH patients of differing severities (61, 62); the simulated patients within NAFLDsym are consistent with these reported data (Fig. 1). The Kupffer cells, macrophages and neutrophils generate a variety of mediators, depending on the various queues presented to them. Within NAFLDsym, these cells can produce TNF-alpha, IL-10, TGF-beta, PDGF, MMP, CCL3, and TIMP. Total mediator production is regulated by the number of viable cells, the ability of each cell type to make each mediator, as well as the cross-regulatory influence of mediators on each other’s production. The levels of each mediator across a range of disease severity in the simulated patients of NAFLDsym are consistent with a variety of different clinical data sets (63–68). Several mediators act on hepatocytes in addition to immune cells, while others participate in regulating fibrotic processes.

The fibrosis sub-model includes the activation and turnover of hepatic stellate cells (HSC) as well as the synthesis and breakdown of collagen. Early in the disease sequelae, the loss of hepatocytes to lipotoxic influences initiates collagen synthesis as part of the wound healing response. Chronic cell loss and ongoing activation of HSC lead to a fibrotic state in NASH patients, which is captured in a subset of the simulated patients of NAFLDsym. HSC are activated via stimulus from TGF-beta, transforming them to a myofibroblast-type state (69–72). The mediator PDGF encourages proliferation of HSC, regulating the number of activated HSC (73). Procollagen synthesis and collagen release by activated HSC are included in NAFLDsym as well as the release of the Pro-C3 fragment that can be used as a circulating biomarker of collagen synthesis rates (74, 75). Similar to the representation in the inflammation sub-model, the total amount of collagen synthesis reflects both the number of activated HSC as well as the propensity for HSC to produce collagen. Collagen synthesis rates are relatively slow (74, 76), and the simulated patients with fibrosis in NAFLDsym are consistent with this observation (Fig. 1). Also participating in the turnover of collagen in the simulated patients with fibrosis are MMP. This group of mediators are responsible for degrading collagen in a multi-step process (77). In addition to being released by macrophages and neutrophils, MMP are also released by activated HSC in the simulated patients (77).

**Fig. 2:**
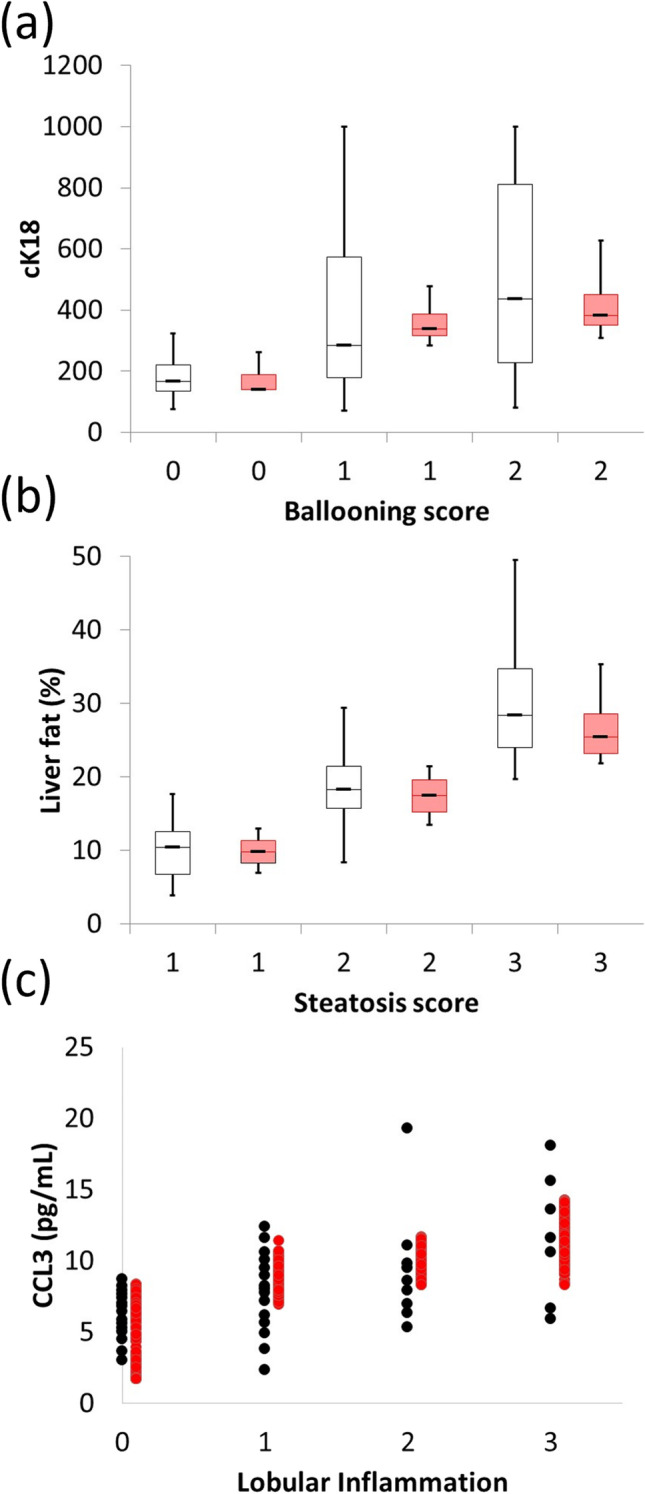
Simulation results and clinical data describing the correspondence between the simulated histologic components of NAS with related outputs. The range of cytokeratin-cleaved K18 (cK18) and histologic ballooning (**a**), liver fat measured by MRI-PDFF and histologic steatosis (**b**), andCCL3 and histologic lobular inflammation scores. Red bars or symbols denote results from simulated patients while black symbols and black bars denote clinical data from Aida *et al*. (**a**), Middleton *et al*. (**b**), and du Plessis *et al*. (**c**).

NAFLDsym simulation outputs include representations of the currently-accepted histologic biomarkers of efficacy for NASH; the NAFLD activity score (NAS) and fibrosis stage (7). The representation of NAS includes the individual components, including steatosis, ballooning, and inflammation histologic (78, 79). These outputs have been validated by comparing with various types of clinical data (56, 80–85) (Fig. 2). Fibrosis stage is also simulated in NAFLDsym; this representation accounts for both the location (centrilobular, midlobular, peripoportal) and quantity of collagen within the liver and is consistent with the histologic scoring paradigm for NASH patients (79, 86). Additional simulation outputs that align with commonly-measured NASH biomarkers include the relative amount of liver fat (consistent with MRI-PDFF measurements), body weight, BMI, plasma ALT, plasma AST, and plasma triglycerides. As mentioned above, the circulating biomarker Pro-C3 is also simulated as an indicator of collagen biosynthesis. It should additionally be pointed out that NAFLDsym does not include several additional combination NASH biomarkers that continue to undergo qualification such as FIB-4, NFS, and ELF (87). These biomarkers could be added in the future as they become qualified.

**Fig. 3:**
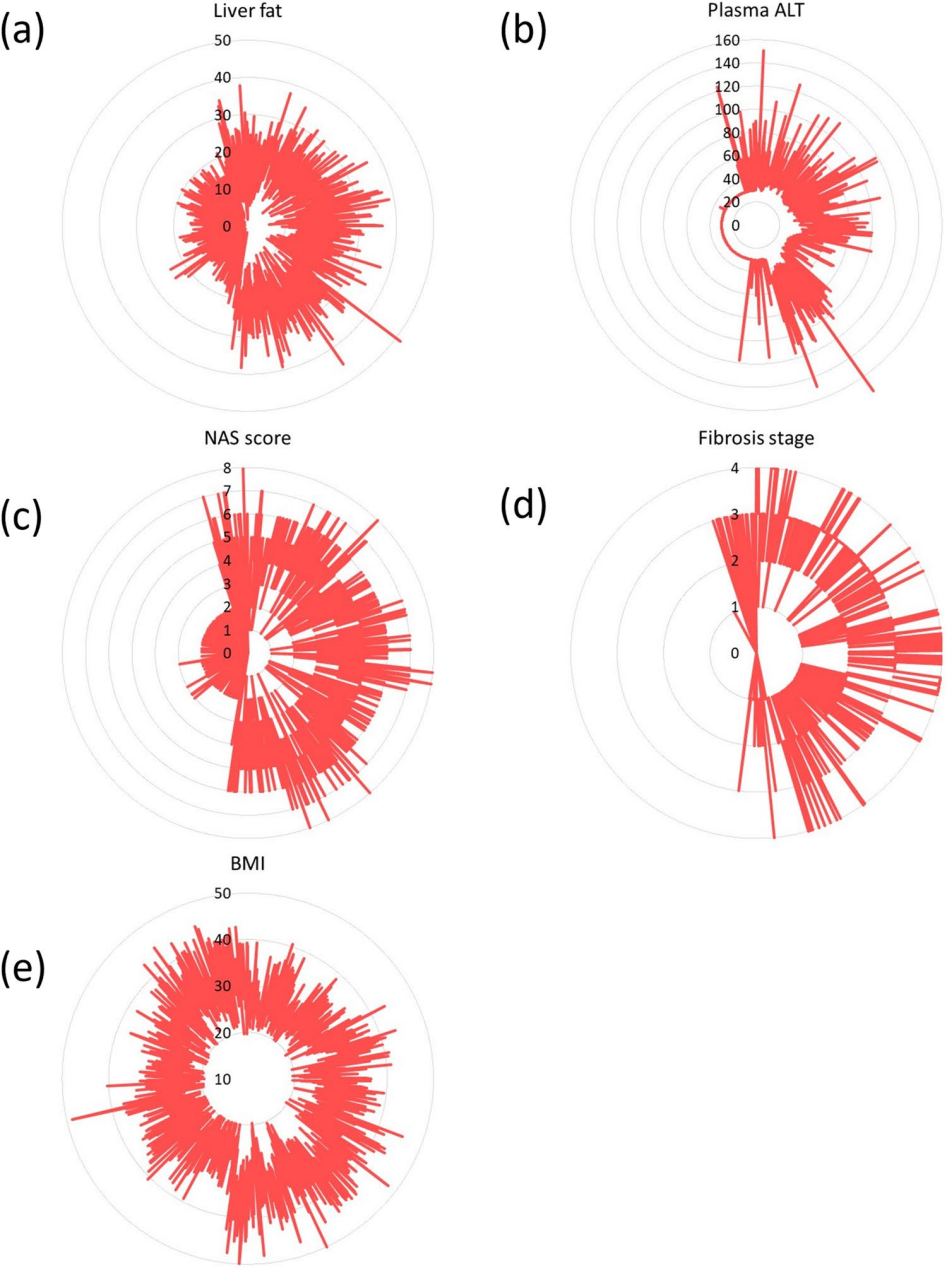
Simulation results for untreated simulated patients within SimPops in NAFLDsym, including liver fat (**a**, units are %), plasma ALT (**b**, units are U/L), NAS (**c**), fibrosis stage (**d**), and BMI (**e**, units are kg/m^2^). Note that each simulated patient retains the same position on each radial plot.

The substantial degree of inter-patient variability amongst NASH and NAFLD patients highlights the utility of the inclusion of > 1700 simulated patients within the NAFLD-NASH SimPops in NAFLDsym. This SimPops includes both mechanistic and clinical between-patient variability. Mechanistic variability is imposed within key aspects of each submodel, including parameters from the following categories across NASH pathophysiology: body weight (55, 88, 89), adipose fatty acid release rates (39), hepatic de novo lipogenesis (40, 41, 90, 91), hepatic VLDL-TG export (47, 48), hepatocellular mitochondrial function (92), hepatic glucose uptake(43, 44), hepatic and extrahepatic plasma TG clearance (55, 89, 93–97), hepatic antioxidant status (52, 98, 99), hepatocellular apoptotic sensitivity to oxidative stress (100), hepatocellular regeneration rates (101, 102), hepatocyte extracellular vesicle release rates (60), macrophage and neutrophil recruitment (80, 85), immune mediator production rates, hepatic stellate cell activation and proliferation (70–72), collagen synthesis and proteolysis rates (32, 33, 36). Parameters and references supporting the selection of these parameters are listed in Table I. Each simulated patient within the SimPops is validated by simultaneous comparison with clinical data across each of these axes. Simulated patients that produce simulation results within the reported ranges of these clinical data boundaries is considered acceptable. Some examples are given in Fig. 1. Inter-patient variability in key clinical measurements emerges from the parametrically-imposed mechanistic variability. Figure 3 illustrates the wide range of variability across several key clinical outputs, including fibrosis stage, NAS, plasma ALT, liver fat, and BMI. The SimPops includes patients with a wide range of NASH patient characteristics (no NASH to severe NASH, no fibrosis to substantial fibrosis, no steatosis to high liver fat, normal plasma ALT to above upper limit of normal). Simulated cohorts (SimCohorts) that include simulated patients with certain clinical characteristics can be selected from the larger SimPops to support simulations of clinical studies at various points in the clinical development pipeline (103).

**Table I:** Summary of Parameters Included in NAFLD SimPops

Parameter Name in NAFLDsym	Data Source for Distribution
Vmax for aHSC proliferation	Assumed standard deviation of ± 20% and parameter range of 2.5 times the S.D. and validated with data from Abdeen 2009, El Gendi 2012, Washington 2000
ATP decrement necrosis Vmax	Assumed standard deviation of ± 20% and parameter range of 2.5 times the S.D. and validated with outcome data
Basal fasting glucose	Browning 2004, Maximos 2015, Copaci 2015, Dudekula 2014, Wong 2013, Stepnova 2010, Tanaka 2013, Zein 2012
Basal plasma triglycerides concentration	Yki-Jarvinen 2014, Maximos 2015
Basal value of mito ETC flux	Perez-Carreras 2003
Rate constant for FFA release from Peripheral storage	Based on relationship between fat mass and adipose fatty acid release described by Mittendorfer 2009
Basal liver triglycerides	Yki-Jarvinen 2014, Maximos 2015, Browning 2004
Body Mass	Yki-Jarvinen 2014, Maximos 2015, Browning 2005
Caspase-mediated apoptosis scaling constant	Bantel 2001
Liver macrophage CCL3 production Vmax	Assumed standard deviation of ± 20% and parameter range of 2.5 times the S.D. and validated with data from DuPlessis 2015, DuPlessis 2016
CL activated HSC apoptosis scalar	El-Gendi 2012, Abdeen 2012, Carpino 2004 (to provide steady state aHSC in accordance with the effects of the CL_aHSC crowding_scalar on aHSC proliferation)
CL activated HSC crowding scalar	El-Gendi 2012, Abdeen 2012, Carpino 2004
CL fibrosis hepatocyte displacement scalar	Carpino 2004, D’Ambrosio 2012
Collagen 1 baseline formation rate	Decaris 2017, Masugi 2018
Collagen 1 formation rate	Decaris 2017, Masugi 2018
Collagen 3 baseline formation rate	Decaris 2017, Masugi 2018
Collagen 3 formation rate	Decaris 2017, Masugi 2018
Extracellular vesicle release from apoptotic cells	Povero 2016
Maximum LSEC HGF production rate per liver LSEC	Assumed standard deviation of ± 20% and parameter range of 2.5 times the S.D. and validated with outcome data
Maximum macrophage HGF production rate per macrophage	Dominguez-Perez 2016, Balaban 2006, Agrawal 2013
Maximum neutrophil HGF production rate per liver neutrophil	Dominguez-Perez 2016, Balaban 2006, Agrawal 2013
HGF mediated regeneration Vmax	Assumed standard deviation of ± 20% and parameter range of 2.5 times the S.D. and validated with outcome data
Vmax for HSC activation	Assumed standard deviation of ± 20% and parameter range of 2.5 times the S.D. and validated with data from Abdeen 2009, El Gendi 2012, Washington 2000
Rate constant for DNL precursor production	Lambert 2014, Donnely 2005, Lee 2015, Diraison 2003
Rate constant for lactate contribution to DNL	Lambert 2014, Donnely 2005, Lee 2015, Diraison 2003
Conversion of mature to labile collagen rate constant	Arima 2004, D’Ambrosio 2012
Rate constant for hepatic Chylo-TG uptake	Tushuizen 2010, McQuaid 2011
Rate constant for hepatic glucose uptake	McMahon 1989, Cersosimo 2011
Rate constant for hepatic VLDL-TG uptake	Yki-Jarvinen 2014, Maximos 2015, Mittendorfer 2003, Sane 1988, Beil 1982
Km for FFA2DAG	Required to have appropriate dynamics with TG Lipolysis mechanism activated
Km for triglyceride lipolysis	Variability in this parameter provides variability in the liver TG-ALT relationship described by Yki-Jarvinen 2014, Maximos 2015, Browning 2004
Rate constant for Chylo-TG uptake by peripheral tissues	Tushuizen 2010, McQuaid 2011
Rate constant for VLDL-TG uptake by peripheral tissues	Yki-Jarvinen 2014, Maximos 2015, Mittendorfer 2003, Sane 1988, Beil 1982
Vmax for LOX	Assumed standard deviation of ± 20% and parameter range of 2.5 times the S.D. and validated with data from Mesarwi 2015
ML fibrosis hepatocyte displacement scalar	Carpino 2004, D’Ambrosio 2012
Vmax for MMP	Assumed standard deviation of ± 20% and parameter range of 2.5 times the S.D
Vmax for MMP (fragments)	Assumed standard deviation of ± 20% and parameter range of 2.5 times the S.D
PP fibrosis hepatocyte displacement scalar	Carpino 2004, D’Ambrosio 2012
Half-life for plasma Pro-C3	Assumed standard deviation of ± 50% and parameter range of 2.5 times the S.D. and validated with data reported by Levin 2017 (abstract)
Procollagen 1 production rate	Decaris 2017, Masugi 2018
Procollagen 1 baseline production rate	Decaris 2017, Masugi 2018
Procollagen 3 production rate	Decaris 2017, Masugi 2018
Procollagen 3 baseline production rate	Decaris 2017, Masugi 2018
Scaling coeff. representing reserve mitochondria function	Perez-Carreras 2003
Liver RNS/ROS baseline clearance Vmax	Hardwick 2010, Videla 2004, Tanaka 2013
Serum adiponectin initial value	Adiels 2006
Prior (weight) for liver TG % to steatosis Grade 0 model	Randomized distribution of histologic steatosis to provide variability between the four grades
Prior (weight) for liver TG % to steatosis Grade 1 model	Randomized distribution of histologic steatosis to provide variability between the four grades
Prior (weight) for liver TG % to steatosis Grade 2 model	Randomized distribution of histologic steatosis to provide variability between the four grades
Prior (weight) for liver TG % to steatosis Grade 3 model	Randomized distribution of histologic steatosis to provide variability between the four grades
Triglyceride lipolysis switch	Required to ensure TG Lipolysis mechanism activated
Liver macrophage TGF-beta production Vmax	Assumed standard deviation of ± 20% and parameter range of 2.5 times the S.D. and validated with data from Das 2011
Maximum inhibition of MMP by TIMPs	Assumed standard deviation of ± 20% and parameter range of 2.5 times the S.D
Liver macrophage TIMP production Vmax	Ando 2018, Miele 2009
Liver macrophage TNF-alpha production Vmax	Assumed standard deviation of ± 20% and parameter range of 2.5 times the S.D. and validated with data from Das 2011, Zahran 2013, Hui 2004, Paredes-Turrubiarte 2016
Vmax for FFA2DAG	Required to ensure appropriate hepatocyte fatty acid and DAG dynamics
VLDL-triglyceride secretion rate Vmax	Fabbrini 2008, Adiels 2006

**Fig. 4:**
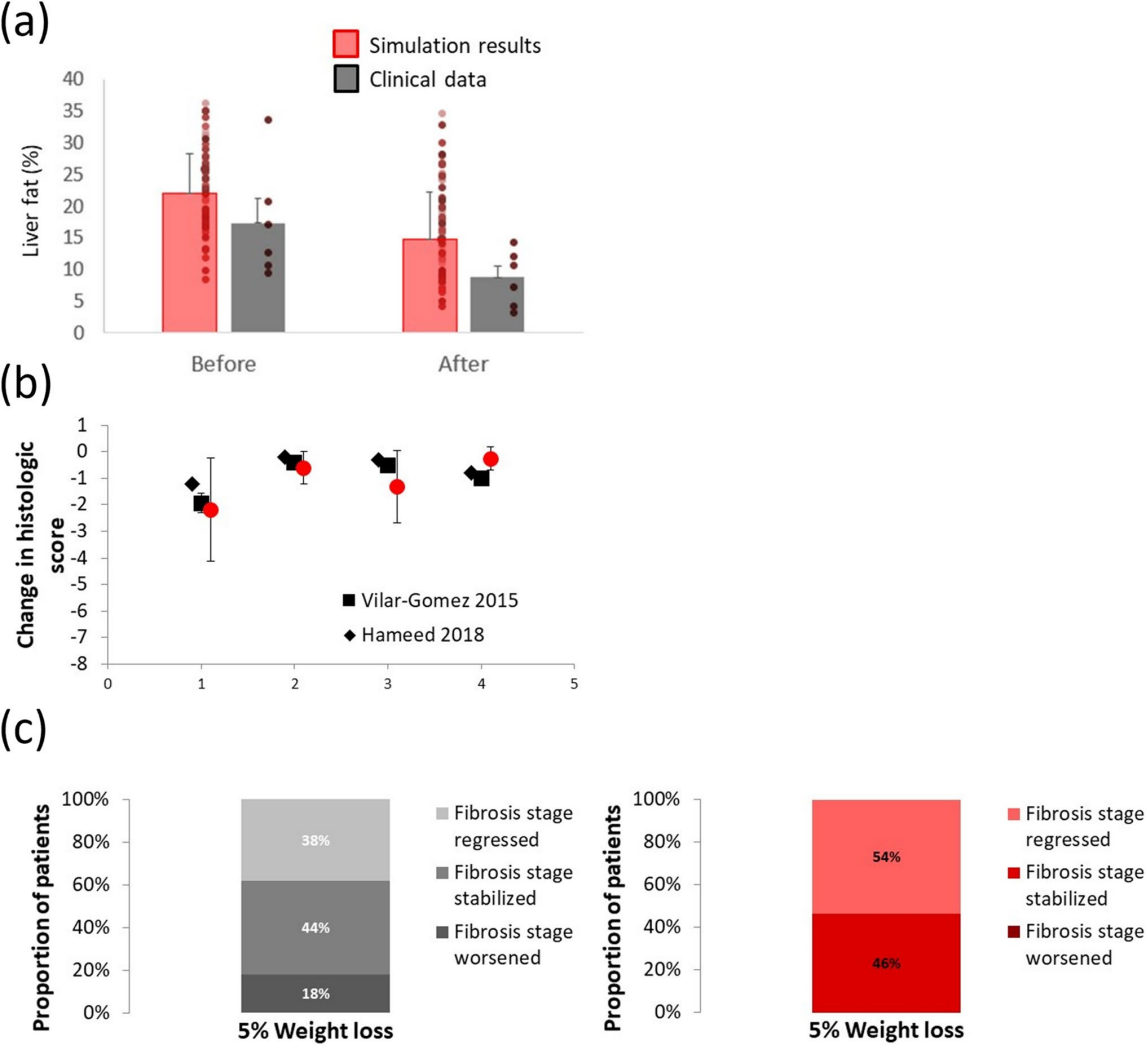
Simulation results and clinical data illustrating the appropriate degree of relief in simulated NASH patients in response to 5–10% weight loss achieved via restriction of caloric intake. Liver fat before and after six months of 10% weight loss, as compared with clinical data from Smith *et al*. Mean responses and individual simulated and clinical patient results displayed (**a**); absolute change in overall NAS and respective components after 5–7% weight loss over 12 months, as compared with clinical data from Vilar-Gomez *et al*. and Hameed *et al*. Note that a negative value indicates reduction relative to initial values (**b**); fraction of patients with worsened, stabilized, or regressed fibrosis stage after 12 months of 5–7% weight loss, as compared with clinical data from Vilar-Gomez *et al*. (**c**). Clinical results are summarized in figure on left, while simulation results are in figure on right Red bars or symbols denote results from SimCohorts while black or gray symbols denote clinical data.

While there has been substantial activity towards developing pharmaceutical treatments for NASH patients, none are currently available. As such, the standard of care for NASH treatment remains weight loss (104–107). Weight loss has been shown to be an efficacious treatment approach, although patient compliance is challenging (107). Consistent with current QSP methodologies, we applied weight loss to our SimPops to further validate the simulated patients (13, 14, 16). Included as a submodel within NAFLDsym is the QSP model of body weight developed by Hall *et al*. (108). As such, NAFLDsym is capable of accurately predicting weight loss and changes in body composition with reduced caloric intake. This submodel mechanistically interacts with the other NAFLDsym sub-models in the following ways: Reduced food intake acta to diminish substrate availability for hepatic de novo lipogenesis and adipose fatty acid release rates are reduced as fat mass is decreased (38, 42, 109). These mechanisms combine to reduce the lipid burden upon the liver, leading to further improvements in the downstream pathophysiology of NASH as well. Figure 4 illustrates the simulation results for SimCohorts selected to align with clinical cohorts for the given studies. Of note is the appropriate simulated reductions in liver fat, NAS (including individual components), and fibrosis stage with 5–10% weight loss via reduced caloric intake over 6–12 months. NAFLDsym has additionally been used to simulate several potential treatments for NASH patients (e.g., anti-FGFR1/KLB bispecific antibody, cenicriviroc), including those that elicited improvements in NASH and others that did not (36, 37). The accurate prediction of the responses to these treatments further validates NAFLDsym and the SimPops.

**Fig. 5:**
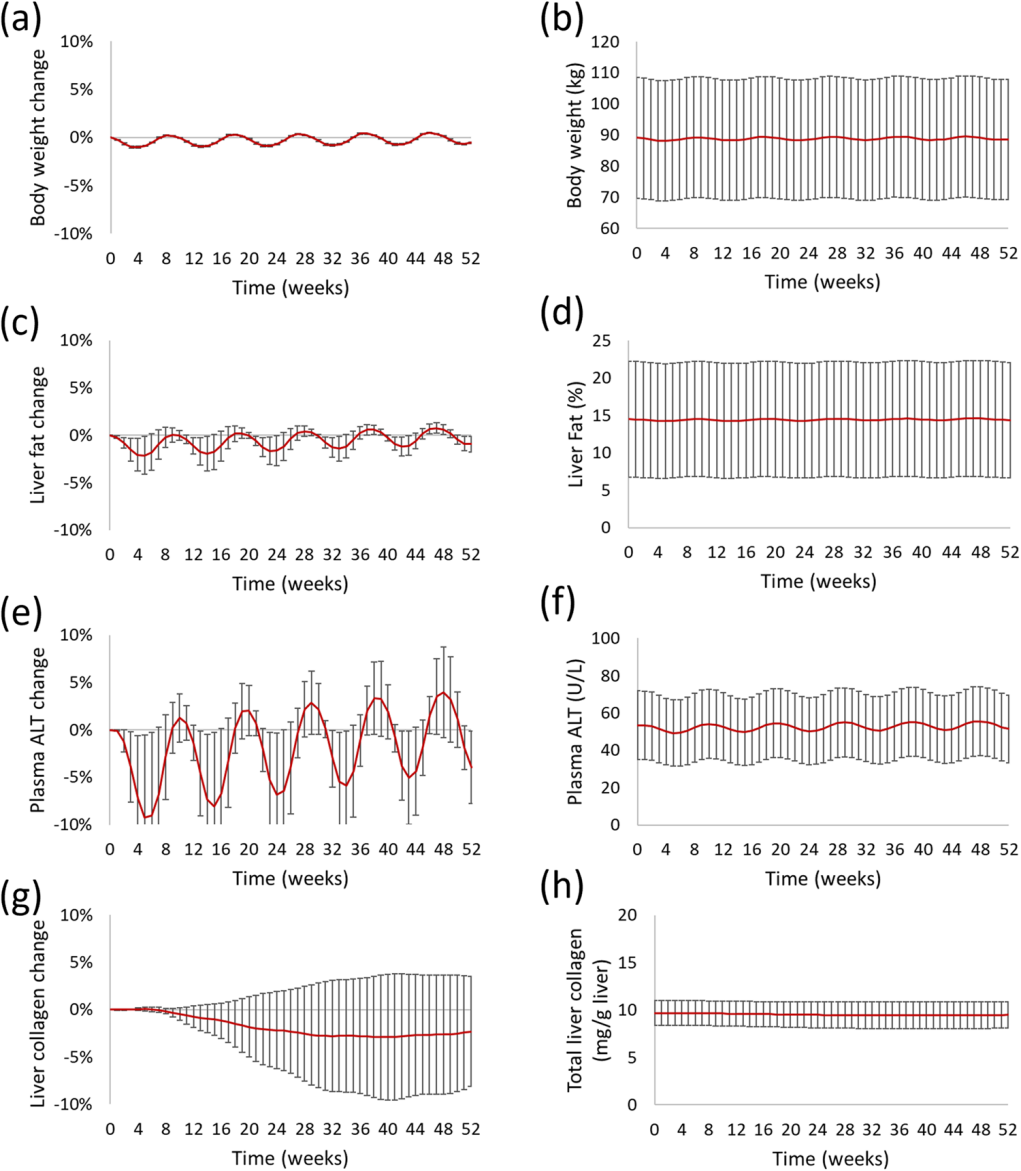
Predicted relative changes (left) in and absolute levels (right) of body weight (**a**, **b**), liver fat (**c**, **d**), plasma ALT (**e**, **f**), and liver collagen (**g**,**h**) in NASH SimCohorts over time due to yo-yo dieting. Mean ± standard deviation plotted for all figures.

NAFLDsym has recently been used to identify potential mechanistic contributors to the relatively high response rate in improved fibrosis stage within placebo cohorts in clinical studies (23). Specifically, NAFLDsym was used to simulate subtle adjustments in food intake over the course of a simulated 52 week study that led to periodic weight loss and weight gain; body weight was not predicted to change appreciably by the end of the simulated study (Fig. 5a, 5b). A simulated cohort of 90 simulated NASH patients was subjected to this so-called yo-yo dieting protocol. The baseline characteristics of the simulated cohort are given in Table II. Food intake was adjusted every four weeks to enable 1% weight gain and weight loss in repeated order. Figure 5 illustrates not only the small changes in body weight over the simulated study, but also the consequent predicted changes in liver fat, plasma ALT, and liver collagen. Both liver fat and plasma ALT are predicted to decline with the reduced food intake (and associated weight loss) and increase with the periodic rebound in body weight. The absolute amounts of body weight, liver fat, and plasma ALT at the conclusion of the 52 week simulated study are quite similar to the values at the beginning of the study. However, liver collagen levels did not follow the same pattern as the other biomarkers. Rather liver collagen was predicted to decline, leading to a predicted reduction in fibrosis stage in 10% of the simulated patients within the cohort (Table III). The notably slow turnover rate of liver collagen (74, 76) prevents liver collagen from having the same periodicity that body weight, liver fat, and plasma ALT do in response to the cyclical adjustments in simulated food intake. These simulation results suggest that, within clinical studies, some proportion of NASH patients in placebo cohorts with reduced fibrosis stage could be experiencing the yo-yo dieting behavioral pattern. Confirmation of these simulation results with additional clinical studies and/or data collection during existing clinical studies could impact the interpretation of data from the placebo cohorts in these studies.

**Table II:** Baseline Simulated Cohort Characteristics

Body weight (kg)	Liver fat (%)	Plasma ALT (U/L)	NAS (score)	Fibrosis score = 2	Fibrosis score = 3
89.1 ± 19.4	17 ± 5	50 ± 12	5.6 ± 3.2	39%	61%

**Table III:** Proportion of Simulated NASH Patients with Predicted Histologic Reductions Over time with Yo-Yo Dieting

	13 weeks	26 weeks	39 weeks	52 weeks
Fibrosis	6%	10%	12%	10%
NAS	4%	0%	0%	0%

## Conclusion

The increasing prevalence of NAFLD and NASH accentuates the need for available treatments in this patient populations. Application of QSP modeling to the development of NASH drug development can help accelerate this process. Moreover, such NASH QSP models should adhere to recommendations put forth by regulatory agencies such as the FDA. These include model transparency and documentation, minimizing complexity of the QSP model, utilizing predefined, quantitative criteria for model validation, as well as the inclusion of useful NASH clinical biomarkers such as NAS and fibrosis stage. Several useful QSP models are currently available to help support NASH drug development to varying extents, with NAFLDsym, in particular, providing the capability of simulating numerous useful aspects of NASH. Ultimately, the partnership between clinical studies and QSP modeling should help provide safe and effective medicines for NASH patients in short order.
